# Ages of Animals

**Published:** 1851-11

**Authors:** 


*Ages of Animals.* — A bear will seldom live beyond twenty years, dogs and wolves live twenty years, a fox fourteen to sixteen. The ordinary age of cats is seventeen years; that of a squirrel, hare or rabbit, five to eight years. Elephants, it is said, live 400 years; the rhinoceros, fifty years; horses may live seventy-two years, but their ordinary age is from twenty-five to thirty years: the chamois live sometimes 100 years. An eagle died at Vienna at the age of 104 years. Ravens live 100; swans 300. A tortoise lived more than 190 years. A sheep seldom lives more than ten, and a cow more than fifteen years. — *Southern Med. and Surg. Journal.*

